# Supplementary material for: Machine Learning Accelerates Crystallization for Structure Determination
Source: Angew Chem Int Ed Engl. 2026 May 4;65(25):e1218503. doi: 10.1002/anie.1218503 (PMC13266958; doi:10.1002/anie.1218503)

checkCIF/PLATON report

Structure factors have been supplied for datablock(s) lcz041_auto

THIS REPORT IS FOR GUIDANCE ONLY. IF USED AS PART OF A REVIEW PROCEDURE FOR PUBLICATION, IT SHOULD NOT REPLACE THE EXPERTISE OF AN EXPERIENCED CRYSTALLOGRAPHIC REFEREE.

No syntax errors found. CIF dictionary Interpreting this report

Datablock: lcz041_auto

Bond precision:	C-C = 0.0477 A		Wavelength=1.54184
Cell:	a=14.7184(4)	b=13.4029(4)	c=18.7533(7)
	alpha=90	beta=90	gamma=90
Temperature:	100 K		

	Calculated	Reported
Volume	3699.5(2)	3699.4(2)
Space group	P n a 21	P n a 21
Hall group	P 2c -2n	P 2c -2n
Moiety formula	C28 H19 Ag3 F18 N6 O5 [+ solvent]	C15 H3 Ag3 F18 N6, C13 H16 O5
Sum formula	C28 H19 Ag3 F18 N6 O5 [+ solvent]	C28 H19 Ag3 F18 N6 O5
Mr	1185.10	1185.10
Dx,g cm-3	2.128	2.128
Z	4	4
Mu (mm-1)	13.910	13.910
F000	2288.0	2288.0
F000’	2298.98	
h,k,lmax	18,16,23	17,16,22
Nref	7398[3818]	5868
Tmin,Tmax	0.107,0.124	0.071,1.000
Tmin’	0.047	

Correction method= # Reported T Limits: Tmin=0.071 Tmax=1.000
AbsCorr = MULTI-SCAN

Data completeness= 1.54/0.79 Theta(max)= 72.972

R(reflections)= 0.1501(5399) wR2(reflections)= 0.3818(5868)

S = 1.776 Npar= 524

The following ALERTS were generated. Each ALERT has the format
test-name_ALERT_alert-type_alert-level.
Click on the hyperlinks for more details of the test.

Alert level A

PLAT971_ALERT_2_A Check Calcd Resid. Dens. 0.76Ang From Ag03 4.73 eA-3

Author Response: This Alert is due to presence of residual density in the presence of heavy metal atom (Ag).

PLAT971_ALERT_2_A Check Calcd Resid. Dens. 0.76Ang From Ag01 4.51 eA-3

Author Response: This Alert is due to presence of residual density in the presence of heavy metal atom (Ag).

PLAT971_ALERT_2_A Check Calcd Resid. Dens. 0.73Ang From Ag02 4.13 eA-3

Author Response: This Alert is due to presence of residual density in the presence of heavy metal atom (Ag).

PLAT971_ALERT_2_A Check Calcd Resid. Dens. 0.97Ang From Ag02 4.12 eA-3

Author Response: This Alert is due to presence of residual density in the presence of heavy metal atom (Ag).

PLAT971_ALERT_2_A Check Calcd Resid. Dens. 0.79Ang From Ag02 3.98 eA-3

Author Response: This Alert is due to presence of residual density in the presence of heavy metal atom (Ag).

PLAT971_ALERT_2_A Check Calcd Resid. Dens. 0.96Ang From Ag01 3.98 eA-3

Author Response: This Alert is due to presence of residual density in the presence of heavy metal atom (Ag).

PLAT971_ALERT_2_A Check Calcd Resid. Dens. 0.97Ang From Ag01   3.95 eA-3

Author Response: This Alert is due to presence of residual density in the presence of heavy metal atom (Ag).

PLAT973_ALERT_2_A Check Calcd Positive Resid. Density on   Ag01   4.14 eA-3

Author Response: This Alert is due to presence of residual density in the presence of heavy metal atom (Ag).

PLAT973_ALERT_2_A Check Calcd Positive Resid. Density on   Ag03   3.76 eA-3

Author Response: This Alert is due to presence of residual density in the presence of heavy metal atom (Ag).

Alert level B

PLAT084_ALERT_3_B High wR2 Value (i.e. > 0.25) ....   0.38 Report

Author Response: Weakly diffracting crystal leading to relatively poor data of challenging sample.

PLAT097_ALERT_2_B Large Reported Max. (Positive) Residual Density   4.82 eA-3

Author Response: This Alert is due to presence of residual density in the presence of heavy metal atom (Ag).

PLAT342_ALERT_3_B Low Bond Precision on C-C Bonds ....   0.04773 Ang.

Author Response: Disordered structure.

PLAT971_ALERT_2_B Check Calcd Resid. Dens. 0.77Ang From Ag03   3.37 eA-3

Author Response: This Alert is due to presence of residual density in the presence of heavy metal atom (Ag).

PLAT971_ALERT_2_B Check Calcd Resid. Dens. 0.84Ang From Ag03   3.23 eA-3

Author Response: This Alert is due to presence of residual density in the presence of heavy metal atom (Ag).

```
PLAT971_ALERT_2_B Check Calcd Resid. Dens.  0.95Ang From Ag03           3.14 eA-3
```

Author Response: This Alert is due to presence of residual density in the presence of heavy metal atom (Ag).

```
PLAT971_ALERT_2_B Check Calcd Resid. Dens.  0.32Ang From Ag02           3.02 eA-3
```

Author Response: This Alert is due to presence of residual density in the presence of heavy metal atom (Ag).

```
PLAT971_ALERT_2_B Check Calcd Resid. Dens.  1.77Ang From Ag03           2.90 eA-3
```

Author Response: This Alert is due to presence of residual density in the presence of heavy metal atom (Ag).

```
PLAT971_ALERT_2_B Check Calcd Resid. Dens.  0.75Ang From Ag02           2.90 eA-3
```

Author Response: This Alert is due to presence of residual density in the presence of heavy metal atom (Ag).

```
PLAT971_ALERT_2_B Check Calcd Resid. Dens.  0.76Ang From Ag01           2.86 eA-3
```

Author Response: This Alert is due to presence of residual density in the presence of heavy metal atom (Ag).

Alert level C

```
DIFMX02_ALERT_1_C  The maximum difference density is > 0.1*ZMAX*0.75
            The relevant atom site should be identified.
PLAT082_ALERT_2_C High R1 Value ................................       0.15 Report
PLAT090_ALERT_3_C Poor Data / Parameter Ratio (Zmax > 18) ........       7.02 Note
PLAT094_ALERT_2_C Ratio of Maximum / Minimum Residual Density ....       2.11 Report
PLAT213_ALERT_2_C Atom F00B            has ADP max/min Ratio .....        3.5 prolat
PLAT220_ALERT_2_C NonSolvent   Resd 1  C   Ueq(max)/Ueq(min) Range        3.1 Ratio
PLAT234_ALERT_4_C Large Hirshfeld Difference F8        --C16      .       0.22 Ang.
PLAT234_ALERT_4_C Large Hirshfeld Difference F00R      --C01G     .       0.24 Ang.
PLAT241_ALERT_2_C High   'MainMol' Ueq as Compared to Neighbors of       C00T Check
PLAT241_ALERT_2_C High   'MainMol' Ueq as Compared to Neighbors of       C01D Check
PLAT241_ALERT_2_C High   'MainMol' Ueq as Compared to Neighbors of       C015 Check
PLAT242_ALERT_2_C Low    'MainMol' Ueq as Compared to Neighbors of       C01A Check
PLAT242_ALERT_2_C Low    'MainMol' Ueq as Compared to Neighbors of       C01N Check
PLAT242_ALERT_2_C Low    'MainMol' Ueq as Compared to Neighbors of       C00V Check
PLAT362_ALERT_2_C Short  C(sp3)-C(sp2) Bond  C00P      - C00U     .       1.32 Ang.
PLAT363_ALERT_2_C Long   C(sp3)-C(sp2) Bond  C012      - C014     .       1.65 Ang.
PLAT369_ALERT_2_C Long   C(sp2)-C(sp2) Bond  C01B      - C01D     .       1.55 Ang.
PLAT412_ALERT_2_C Short Intra XH3 .. XHn     H01F      ..H01O     .       1.89 Ang.
                                                      x,y,z  =      1_555 Check
```

```
PLAT906_ALERT_3_C Large K Value in the Analysis of Variance ......      2.763 Check
PLAT911_ALERT_3_C Missing FCF Refl Between Thmin & STh/L=    0.600          5 Report
                4  0  0,  13  7  0,  10 13  3,  17  2  5,   0  0 12,
PLAT971_ALERT_2_C Check Calcd Resid. Dens.  0.93Ang From N00M           2.22 eA-3
```

Author Response: This Alert is due to presence of residual density in the presence of heavy metal atom (Ag).

```
PLAT971_ALERT_2_C Check Calcd Resid. Dens.  0.91Ang From N00H           2.19 eA-3
```

Author Response: This Alert is due to presence of residual density in the presence of heavy metal atom (Ag).

```
PLAT971_ALERT_2_C Check Calcd Resid. Dens.  0.87Ang From C01C           1.58 eA-3
```

Author Response: This Alert is due to presence of residual density in the presence of heavy metal atom (Ag).

```
PLAT971_ALERT_2_C Check Calcd Resid. Dens.  0.72Ang From C01A           1.52 eA-3
```

Author Response: This Alert is due to presence of residual density in the presence of heavy metal atom (Ag).

```
PLAT972_ALERT_2_C Check Calcd Resid. Dens.  1.40Ang From Ag02          -2.36 eA-3
PLAT972_ALERT_2_C Check Calcd Resid. Dens.  1.14Ang From N00M          -2.24 eA-3
PLAT972_ALERT_2_C Check Calcd Resid. Dens.  1.34Ang From Ag03          -2.14 eA-3
PLAT972_ALERT_2_C Check Calcd Resid. Dens.  0.87Ang From N00M          -2.11 eA-3
PLAT972_ALERT_2_C Check Calcd Resid. Dens.  0.78Ang From N00O          -2.03 eA-3
PLAT972_ALERT_2_C Check Calcd Resid. Dens.  1.33Ang From Ag02          -1.90 eA-3
PLAT972_ALERT_2_C Check Calcd Resid. Dens.  1.16Ang From N00O          -1.86 eA-3
PLAT972_ALERT_2_C Check Calcd Resid. Dens.  1.42Ang From Ag03          -1.85 eA-3
PLAT972_ALERT_2_C Check Calcd Resid. Dens.  1.38Ang From Ag02          -1.85 eA-3
PLAT972_ALERT_2_C Check Calcd Resid. Dens.  1.44Ang From Ag03          -1.83 eA-3
PLAT972_ALERT_2_C Check Calcd Resid. Dens.  1.58Ang From Ag03          -1.73 eA-3
PLAT972_ALERT_2_C Check Calcd Resid. Dens.  1.25Ang From Ag03          -1.64 eA-3
PLAT972_ALERT_2_C Check Calcd Resid. Dens.  1.53Ang From Ag02          -1.63 eA-3
PLAT977_ALERT_2_C Check Negative Difference Density on H00X      .     -0.59 eA-3
PLAT977_ALERT_2_C Check Negative Difference Density on H01C      .     -0.40 eA-3
PLAT977_ALERT_2_C Check Negative Difference Density on H01E      .     -0.43 eA-3
PLAT977_ALERT_2_C Check Negative Difference Density on H01G      .     -0.77 eA-3
PLAT977_ALERT_2_C Check Negative Difference Density on H01I      .     -0.42 eA-3
```

Alert level G

```
PLAT002_ALERT_2_G Number of Distance or Angle Restraints on AtSite          2 Note
PLAT003_ALERT_2_G Number of Uiso or U(i,j)  Restrained non-H-Atoms         40 Report
PLAT042_ALERT_1_G Calc. and Reported MoietyFormula Strings  Differ     Please Check
            Calc: C28 H19 Ag3 F18 N6 O5
            Rep.: C15 H3 Ag3 F18 N6, C13 H16 O5
PLAT072_ALERT_2_G SHELXL First  Parameter in WGHT  Unusually Large       0.20 Report
```

```
PLAT172_ALERT_4_G The CIF-Embedded .res File Contains DFIX Records          1 Report
PLAT177_ALERT_4_G The CIF-Embedded .res File Contains DELU Records          6 Report
PLAT178_ALERT_4_G The CIF-Embedded .res File Contains SIMU Records          6 Report
PLAT186_ALERT_4_G The CIF-Embedded .res File Contains ISOR Records         11 Report
PLAT188_ALERT_3_G A Non-default SIMU Restraint Value has been used     0.0200 Report
PLAT242_ALERT_2_G Low    'MainMol' Ueq as Compared to Neighbors of       C00P Check
PLAT242_ALERT_2_G Low    'MainMol' Ueq as Compared to Neighbors of       C00W Check
PLAT242_ALERT_2_G Low    'MainMol' Ueq as Compared to Neighbors of       C011 Check
PLAT242_ALERT_2_G Low    'MainMol' Ueq as Compared to Neighbors of        C16 Check
PLAT242_ALERT_2_G Low    'MainMol' Ueq as Compared to Neighbors of       C01G Check
PLAT398_ALERT_2_G Deviating  C-O-C   Angle From 120 for O00Q     .      107.1 Degree
PLAT605_ALERT_4_G Largest Solvent Accessible VOID in the Structure         12 A**3
PLAT720_ALERT_4_G Number of Unusual/Non-Standard Labels ..........         75 Note
              Ag01    Ag02    Ag03    O004    F005    F006    F007    F008
              O00A    F00B    F00C    F00D    F00F    F00G    N00H    N00I
              F00J    F00K    F00L    N00M    O00N    N00O    C00P    O00Q
              F00R    C00S    H00S    C00U    N009    N00E    C01C    C00T
              H00T    C00W    C00X    H00X    F00Y    O010    C011    C012
              H01A    H01B    F013    C014    C018    C019    H01C    H01D
              H01E    C01A    C01B    C01E    H01F    C01D    C01N    H01N
              C015    H015    C00V    C016    C01F    C01G    C01I    H01G
              H01H    H01I    C01J    H01J    H01K    C01L    C01O    C01P
              H01L    H01M    H01O
PLAT860_ALERT_3_G Number of Least-Squares Restraints ............         293 Note
PLAT868_ALERT_4_G ALERTS Due to the Use of _smtbx_masks Suppressed          ! Info
PLAT912_ALERT_4_G Missing # of FCF Reflections Above STh/L=  0.600        118 Note
PLAT913_ALERT_3_G Missing # of Very Strong Reflections in FCF ....          1 Note
                4  0  0,
PLAT915_ALERT_3_G No Flack x Check Done: Low Friedel Pair Coverage         61 %
PLAT933_ALERT_2_G Number of HKL-OMIT Records in Embedded .res File          5 Note
               -4  0 -2,  -2  0 -1,  -1 -2 -6,  -1 -2 -5,   0  0-12,
PLAT941_ALERT_3_G Average HKL Measurement Multiplicity ..........         3.2 Low
PLAT969_ALERT_5_G The 'Henn et al.' R-Factor-gap value ..........       9.802 Note
              Predicted wR2: Based on SigI**2  3.90 or SHELX Weight 21.50
PLAT978_ALERT_2_G Number C-C Bonds with Positive Residual Density.          0 Info
```

```
   9 ALERT level A = Most likely a serious problem - resolve or explain
  10 ALERT level B = A potentially serious problem, consider carefully
  42 ALERT level C = Check. Ensure it is not caused by an omission or oversight
  26 ALERT level G = General information/check it is not something unexpected

   2 ALERT type 1 CIF construction/syntax error, inconsistent or missing data
  64 ALERT type 2 Indicator that the structure model may be wrong or deficient
  10 ALERT type 3 Indicator that the structure quality may be low
  10 ALERT type 4 Improvement, methodology, query or suggestion
   1 ALERT type 5 Informative message, check
```

It is advisable to attempt to resolve as many as possible of the alerts in all categories. Often the minor alerts point to easily fixed oversights, errors and omissions in your CIF or refinement strategy, so attention to these fine details can be worthwhile. It is up to the individual to critically assess their own results and, if necessary, seek expert advice.

Validation response form

Please find below a validation response form (VRF) that can be filled in and pasted into your CIF.

```
# start Validation Reply Form
_vrf_DIFMX02_lcz041_auto
;
PROBLEM: The maximum difference density is > 0.1*ZMAX*0.75
RESPONSE: ...
;
_vrf_PLAT082_lcz041_auto
;
PROBLEM: High R1 Value ..................................       0.15 Report
RESPONSE: ...
;
_vrf_PLAT090_lcz041_auto
;
PROBLEM: Poor Data / Parameter Ratio (Zmax > 18) ........       7.02 Note
RESPONSE: ...
;
_vrf_PLAT094_lcz041_auto
;
PROBLEM: Ratio of Maximum / Minimum Residual Density ....       2.11 Report
RESPONSE: ...
;
_vrf_PLAT213_lcz041_auto
;
PROBLEM: Atom F00B            has ADP max/min Ratio .....        3.5 prolat
RESPONSE: ...
;
_vrf_PLAT220_lcz041_auto
;
PROBLEM: NonSolvent   Resd 1  C   Ueq(max)/Ueq(min) Range        3.1 Ratio
RESPONSE: ...
;
_vrf_PLAT234_lcz041_auto
;
PROBLEM: Large Hirshfeld Difference F8       --C16      .       0.22 Ang.
RESPONSE: ...
;
_vrf_PLAT241_lcz041_auto
;
PROBLEM: High   'MainMol' Ueq as Compared to Neighbors of       C00T Check
RESPONSE: ...
;
_vrf_PLAT242_lcz041_auto
;
PROBLEM: Low    'MainMol' Ueq as Compared to Neighbors of       C01A Check
RESPONSE: ...
;
_vrf_PLAT362_lcz041_auto
;
PROBLEM: Short  C(sp3)-C(sp2) Bond  C00P     - C00U     .       1.32 Ang.
RESPONSE: ...
;
_vrf_PLAT363_lcz041_auto
;
```

```
PROBLEM: Long   C(sp3)-C(sp2) Bond  C012     - C014     .       1.65 Ang.
RESPONSE: ...
;
_vrf_PLAT369_lcz041_auto
;
PROBLEM: Long   C(sp2)-C(sp2) Bond  C01B     - C01D     .       1.55 Ang.
RESPONSE: ...
;
_vrf_PLAT412_lcz041_auto
;
PROBLEM: Short Intra XH3 .. XHn     H01F     ..H01O     .       1.89 Ang.
RESPONSE: ...
;
_vrf_PLAT906_lcz041_auto
;
PROBLEM: Large K Value in the Analysis of Variance ......      2.763 Check
RESPONSE: ...
;
_vrf_PLAT911_lcz041_auto
;
PROBLEM: Missing FCF Refl Between Thmin & STh/L=    0.600          5 Report
RESPONSE: ...
;
_vrf_PLAT972_lcz041_auto
;
PROBLEM: Check Calcd Resid. Dens.  1.40Ang From Ag02           -2.36 eA-3
RESPONSE: ...
;
_vrf_PLAT977_lcz041_auto
;
PROBLEM: Check Negative Difference Density on H00X      .      -0.59 eA-3
RESPONSE: ...
;
# end Validation Reply Form
```

PLATON version of 17/09/2025; check.def file version of 17/09/2025

---

duplicate check

No duplication found

Datablock lcz041_auto - ellipsoid plot

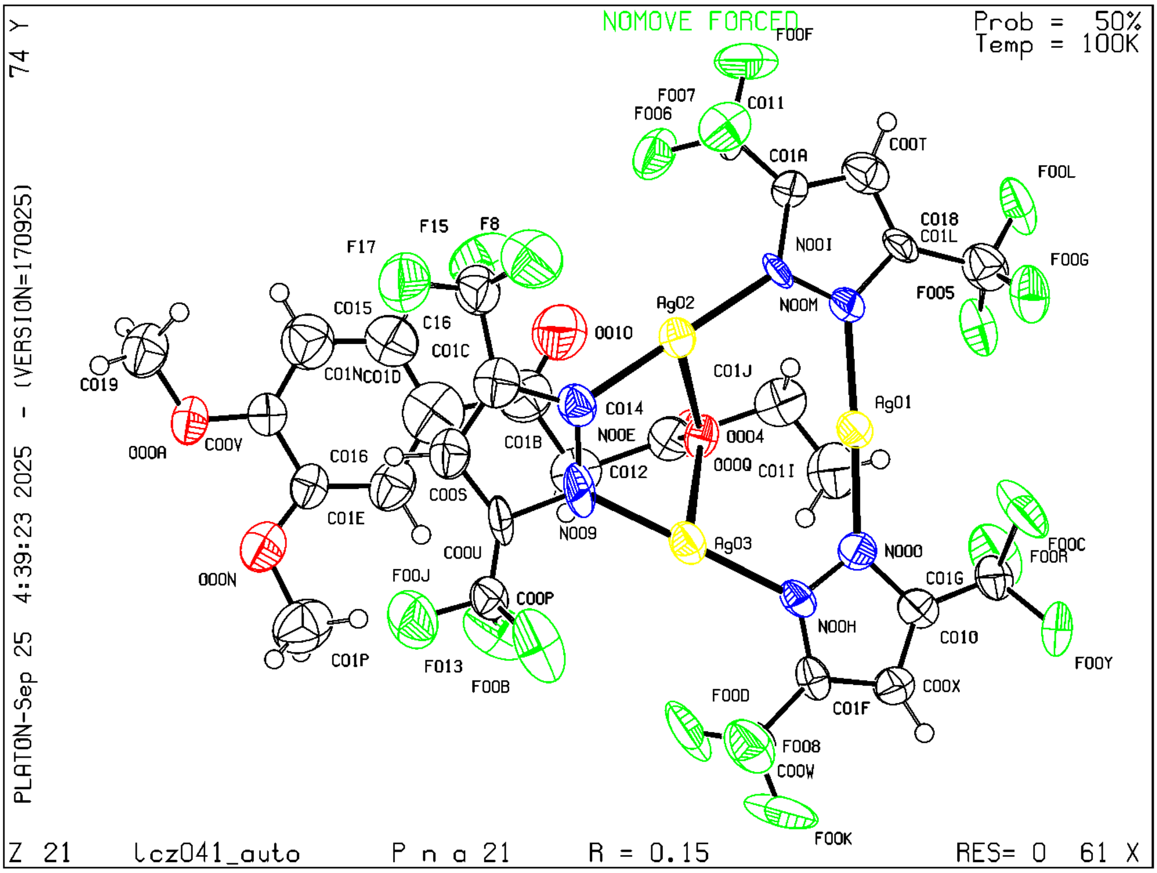

Supplement: Supplementary file 2 — Supporting File 2: anie72462‐sup‐0002‐DataFile.zip. [file ANIE-65-e1218503-s002.zip › supplementary_materials_crystallographic_data submit.zip!/cif_and_checkcif(1-114)/Ag3Pz3_36/Ag3Pz3_36_checkcif.pdf]
